# Solution-printed organic semiconductor blends exhibiting transport properties on par with single crystals

**DOI:** 10.1038/ncomms9598

**Published:** 2015-11-23

**Authors:** Muhammad R. Niazi, Ruipeng Li, Er Qiang Li, Ahmad R. Kirmani, Maged Abdelsamie, Qingxiao Wang, Wenyang Pan, Marcia M. Payne, John E. Anthony, Detlef-M. Smilgies, Sigurdur T. Thoroddsen, Emmanuel P. Giannelis, Aram Amassian

**Affiliations:** 1 Division of Physical Science and Engineering, King Abdullah University of Science and Technology, Thuwal 23955-6900, Saudi Arabia; 2 Advanced Imaging and Characterization Laboratory, King Abdullah University of Science and Technology, Thuwal 23955-6900, Saudi Arabia; 3 Department of Materials Science and Engineering, Cornell University, Ithaca, 14850 New York, USA; 4 Department of Chemistry, University of Kentucky, Lexington, 40506 Kentucky, USA; 5 Cornell High Energy Synchrotron Source, Cornell University, Ithaca, 14850 New York, USA

## Abstract

Solution-printed organic semiconductors have emerged in recent years as promising contenders for roll-to-roll manufacturing of electronic and optoelectronic circuits. The stringent performance requirements for organic thin-film transistors (OTFTs) in terms of carrier mobility, switching speed, turn-on voltage and uniformity over large areas require performance currently achieved by organic single-crystal devices, but these suffer from scale-up challenges. Here we present a new method based on blade coating of a blend of conjugated small molecules and amorphous insulating polymers to produce OTFTs with consistently excellent performance characteristics (carrier mobility as high as 6.7 cm^2^ V^−1^ s^−1^, low threshold voltages of<1 V and low subthreshold swings <0.5 V dec^−1^). Our findings demonstrate that careful control over phase separation and crystallization can yield solution-printed polycrystalline organic semiconductor films with transport properties and other figures of merit on par with their single-crystal counterparts.

## Abstract (web summary)

There continues to be interest in solution processed organic semiconductor thin films exhibiting transport properties on par with single crystals. Here, Niazi *et al*. combine solution-printing of a small molecule organic semiconductor with insulating polymer blending and solvent mixtures to demonstrate hole mobility up to 6.7 cm^2^ V^−1^ s^−1^.

## Graphical abstract

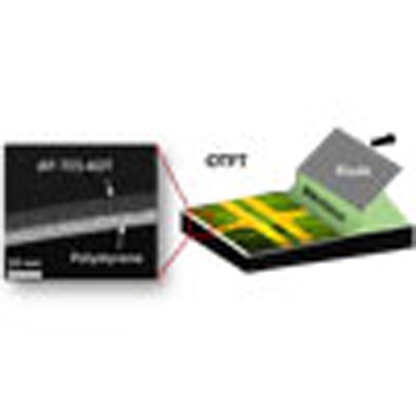

Organic thin-film transistors (OTFTs) have attracted tremendous attention for their potential use in flexible flat panel displays, sensors, storage devices, radio frequency tags and logic circuits^1^,^2^,^3^,^4^,^5^,^6^. Organic semiconductors (OSCs) are especially interesting owing to their compatibility with solution-based coating techniques, which promise low-cost manufacturing by high-throughput continuous roll-to-roll printing methods on flexible substrates^7^. Efforts in the past decade to carefully engineer the molecular structure of soluble OSCs and control their microstructure and morphology have led to carrier mobilities now far surpassing those of amorphous silicon—the current industrial standard TFT material^8^,^9^,^10^, and have been shown to achieve mobilities even in excess of 10 cm^2^ V^−1^ s^−1^ (refs 11, 12). As such, state-of-the-art solution-processed OSCs significantly surpass other p-type solution-processed semiconductors, such as metal oxides and pseudohalides^13^,^14^,^15^,^16^, making OSCs excellent candidates to complement high-performance n-type oxide semiconductors in future solution-processed complementary metal-oxide-semiconductor (CMOS) circuits^17^.

These applications call for a range of performance prerequisites, including high-current on–off ratio, low turn-on and threshold voltages as well as low subthreshold swing^18^. They also require excellent performance yield, reproducibility and uniformity over large areas, which puts significant stress on the solution-manufacturing process of OSCs. Transport in small-molecule OSCs has been shown to be strongly coupled with the crystalline quality and texture purity of lamellar stacks exhibiting two-dimensional in-plane π-stacking, and requires excellent continuity of the OSC thin film with closed domain and grain boundaries^19^,^20^,^21^,^22^. We have recently demonstrated a two-order magnitude modulation in mobility by altering the drying kinetics, which influenced the nucleation and growth of the small-molecule OSC^19^,^23^. Similar performance improvements have been realized in the case of 6,13-bis(triisopropylsilylethynyl) (TIPS) pentacene films by controlling the crystallization with the use of high-boiling solvent additives^24^,^25^. Rapid crystallization of OSCs in processes such as spin coating can lead to extreme sensitivity to drying kinetics and to a lack of the control over nucleation and growth of crystals^26^,^27^. Single crystals of small-molecule OSCs have been therefore found favour in this area and have typically yielded the highest carrier mobility owing to their low-defect nature^28^. Recently, a large-area compatible inkjet process has yielded single crystals of OSC with average hole mobility of 16 cm^2^ V^−1^ s^−1^ and maximum reaching to 31 cm^2^ V^−1^ s^−1^ (ref. 29). However, high throughput and controlled growth of single-crystal OSCs over large-area flexible substrates remain a significant challenge^30^,^31^. Therefore, the device performance reproducibility and uniformity over large substrate areas have been limited by current challenges in solution processing^32^,^33^.

Blending small-molecule OSCs with amorphous semiconducting or insulating polymers has recently been demonstrated as a successful route for manufacturing high-performance OTFTs with high-reproducibility and low-performance spread^34^,^35^. The polymer acts as a binder and helps to overcome the common dewetting challenges associated with small-molecule OSC processing and achieve good uniformity of device performance over large areas^36^,^37^. Vertical stratification in polymer:OSC blends is mainly affected by surface energies of the OSC, polymer, substrate and solvent, by the solubility and miscibility of each component, as well as by the evaporation rate of the solvent^38^. Efforts have therefore been made to optimize morphology, lamellar quality and thin-film crystallinity from insulating polymer:OSC blends using polymers such as polystyrene (PS) and poly(alpha-methyl styrene) (PαMS) under a wide range of processing conditions, including different blend ratios, concentrations, solvents and solvent mixtures, post-thermal treatments, as well as molecular weight (Mw) of the polymer binder^35^,^39^,^40^,^41^,^42^,^43^,^44^,^45^,^46^,^47^,^48^. These attempts have focused exclusively on spin coating and drop casting and yielded performance up to 1.5 cm^2^ V^−1^s^−1^ (ref. 41). While both methods can be scaled to reasonably large substrate sizes, they are incompatible with continuous roll-to-roll processing. In the case of spin-cast OSC: semiconducting polymer blends, top-gate bottom-contact OFETs with mobilities as high as 2.4 and 5.6 cm^2^ V^−1^ s^−1^ have been achieved by blending 2,8-difluoro-5, 11-bis(triethylsilylethynyl) anthradithiophene (diF-TES-ADT) with poly(dimethyl-triarylamine) (PTAA) or poly(dialkyl-fluorene-co-dimethyl-triarylamine) (PFTAA), respectively^49^. Cross-sectional energy filtered transmission electron microscopy (EFTEM) revealed the blend stratifies into a bilayer with the diF-TES-ADT forming a crystalline layer at the air interface. This makes the top-gate device architecture a viable option owing to the low intrinsic mobilities of the PTAA and PFTAA. This performance is close to the single-crystal mobility of the diF-TES-ADT OSC of 6 cm^2^ V^−1^ s^−1^, highlighting the method's remarkable success at bridging the performance gap between thin film and single-crystal devices^50^. The common problem with existing OSC:polymer blend OTFTs is that processing typically occurs via spin coating, a process which is highly wasteful of expensive semiconductor and is not compatible with continuous high-throughput roll-to-roll manufacturing.

Blade-coating and related methods, such as zone casting, knife coating, wire bar coating, and doctor blading have recently demonstrated high-performance OTFTs with minimal material wastage and are compatible with high throughput and continuous roll-to-roll manufacturing^11^,^33^,^51^,^52^,^53^,^54^,^55^,^56^,^57^. These methods have been shown to induce large-scale directional crystallization as well as to induce polymorphism during OSC crystallization, making them potentially significant methods for large-scale manufacturing of organic electronic devices^11^^,^^51^,^58^,^59^,^60^,^61^,^62^. We take the position that blade coating of OSC:polymer blends can yield remarkable performance while mitigating many of the challenges associated to solution processing of small-molecule OSCs.

In this article, we demonstrate high-performance bottom-contact bottom-gate (BCBG) OTFTs prepared via blade coating of a OSC:polymer blend. The amorphous insulating polymer (PS or PαMS) was blended with the small-molecule OSC diF-TES-ADT. We begin by systematically investigating the roles of Mw of the insulating polymer, solution concentration and blading speed on the microstructure, morphology and phase separation of the blends as well as on transport properties. In doing so, we demonstrate OTFTs with carrier mobility as high as 4.5 cm^2^ V^−1^ s^−1^, with on–off ratio exceeding 10^6^, low threshold voltage (<1 V) and low subthreshold swing (<0.5 V dec^−1^), and in many ways surpassing the previous performance records set for this material by spin coating the OSC with a semiconducting polymer in a more complicated top-gate device configuration^49^. The presence of even small amounts of insulating polymer is shown to greatly impact the figures of merit of OTFTs. We then introduce a mixture of solvents with different polarities and achieve a further substantial increase of the carrier mobility up to 6.7 cm^2^ V^−1^ s^−1^ by improving the film quality, surpassing all mobility reports for this material either neat, in a blend or as single crystal^41^,^49^,^50^. We show that this methodology can be used to fabricate high-performance OTFTs with small performance variability and consistently high mobility over a wide range of processing conditions, making this strategy potentially suitable for large-area manufacturing of electronic circuits.

## Results

### Blade-coated polymer-molecule-blend organic thin-film transistors

**Figure 1:**
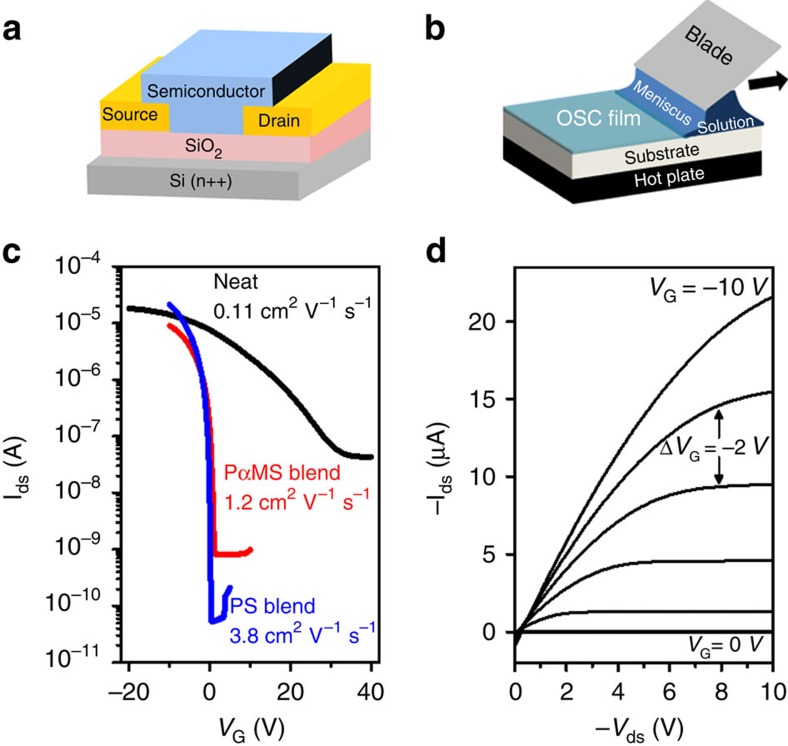
Bottom contact bottom gate (BCBG) OTFTs solution-printed by the blade coating method. (**a**) BCBG device architecture and (**b**) blade-coating set-up. (**c**) Transfer characteristics of OTFTs prepared using neat diF-TES-ADT, diF-TES-ADT: PαMS (100 kDa) and diF-TES-ADT:PS (123 kDa) blends at a blading speed of 1.5 mm s^−1^ and a stage temperature of 70 °C. We employed *V*_ds_=−10 V for blends and *V*_ds_=−20 V for neat OSC. (**d**) Output characteristics of the diF-TES-ADT:PS (123 kDa) device.

The blading speed is known to be a critical parameter for controlling film formation, with low speed yielding ribbon formation while high blading speed leading to spherulite formation^14^,^19^. We fabricated BCBG devices based on neat diF-TES-ADT and 1:1 blends of diF-TES-ADT with PS (123 kDa) and PαMS (100 kDa) for blade-coating speeds ranging between 0.5 and 2 mm s^−1^. Polarized optical micrographs (POMs) of the films in conditions of low (0.5 mm s^−1^) and high (1.5 mm s^−1^) blading speeds are shown in Supplementary Fig. 1, confirming the ribbon formation at low speed and more of a spherulitic microstructure in conditions of high blading speed. In Fig. 1a, we show the BCBG architecture consisting of Si as gate, SiO_2_ as gate dielectric and thermally evaporated Au used as source and drain contacts. The schematic in Fig. 1b illustrates the blade-coating process, wherein a blade placed at a shallow angle over the substrate placed on a hot plate entrains the solution in its path, leading to crystalline thin-film formation as the solvent dries. In Fig. 1d, we show the output characteristics of the best performing OTFTs based on diF-TES-ADT:PS (123 kDa) with hole mobility as high as 3.8 cm^2^ V^−1^ s^−1^. As a reference, we have plotted in Supplementary Fig. 2, the gate voltage dependence of hole mobility for devices fabricated on ultraviolet ozone- and O_2_ plasma-treated BCBG substrates. It can be seen that devices prepared by O_2_ plasma treatment show ideal transfer characteristics and reliable mobility extraction^18^. In contrast, for ultraviolet ozone-treated devices, we observe non-ideal transfer characteristics with strong gate voltage-dependent mobility. In Fig. 1c, we depict the transfer characteristics of neat diF-TES-ADT (black), as well as diF-TES-ADT:PαMS (red) and diF-TES-ADT:PS (blue) blends prepared in identical conditions (1.5 mm s^−1^) while gate leakage current is highlighted in Supplementary Fig. 3. The data highlight major differences in hole mobility, threshold voltage, on/off current ratio and subthreshold swing.

**Figure 2:**
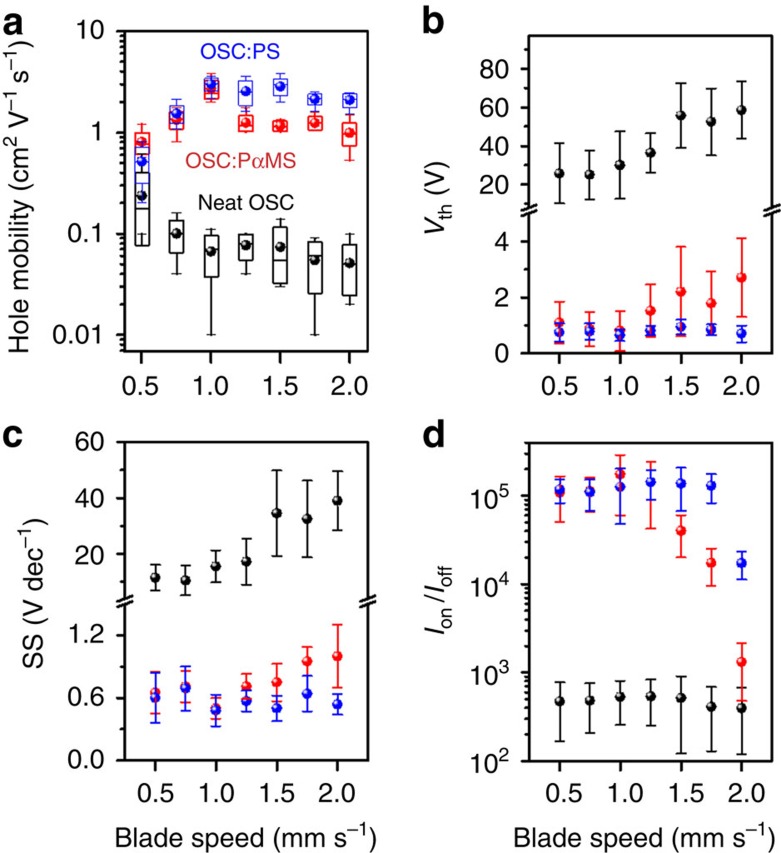
Figures of merit for neat OSC and OSC:polymer blend OTFTs. (**a**) Saturation hole mobility, *μ*, (box and whisker plot) (**b**) threshold voltage, *V*_th_, (**c**) subthreshold swing, SS, and (**d**) *I*_on_/*I*_off_. Error bars in **b**,**c** and **d** represent s.d. from average values.

These differences call for further exploration of the coating speed window (0.5–2.0 mm s^−1^). In Fig. 2a, we have plotted the carrier mobility as a function of blading speed for neat diF-TES-ADT, as well as its blends with PαMS (100 kDa) and PS (123 kDa). In the low-speed regime (0.5 mm s^−1^) blends achieve ∼3–4 times higher mobility than neat samples. In the high-speed regime (1.5 mm s^−1^), the difference is even greater, surpassing an order of magnitude, while the device performance spread is substantially reduced by the use of the blending approach, as indicated by smaller error bars. The threshold voltage (*V*_th_) (Fig. 2b) for neat devices is positive for all speeds, ranging from 10 V at low speed to 70 V at higher speeds. Similarly, the subthreshold swing (SS) increases from 7 V dec^−1^ at low speed to 50 V dec^−1^ at high speed. Blending with the insulating polymer substantially reduces both *V*_th_ and SS to the range of 0.1–4 V and 0.35–1 V dec^−1^, respectively, over the entire blading speed range. This highlights the remarkable benefits of blending the OSC with an insulating polymer and its apparent ability to reduce the interfacial trap state density typically responsible for elevated SS and *V*_th_ values in neat devices. As shown in Table 1, the interfacial trap state density, *N*_it_, where 
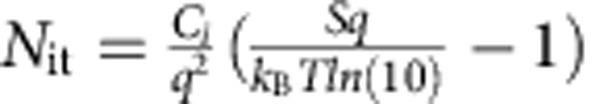
, with *C*_*i*_ the capacitance per unit area of the dielectric, *q* the elementary charge, *S* the subthreshold slope, *k*_B_ the Boltzmann constant and *T* the temperature, decreases by one to two orders of magnitude from ∼10^13^ eV^−1^ cm^2^ to ∼5 × 10^11^ eV^−1^ cm^2^ by the virtue of blending an insulating polymer with the OSC. In the high-speed regime, we also systematically observe PS-based blends outperforming PαMS-based blends in terms of all figures of merit (Fig. 2a–d), a trend we later confirm to hold for a wide range of Mws of the polymer as well. Both polymers are equally soluble in toluene and exhibit very similar Flory–Huggins parameters (∼0.4) (ref. 63), we therefore expect the thermodynamic driving forces for phase separation to be nearly identical. However, the performance of PαMS-based devices produced at a blade speed >1 mm s^−1^ suffers with respect to PS-based devices. Looking more closely at the atomic force micrograph (AFM) of blends produced at 2 mm s^−1^ (Supplementary Fig. 4), we find the topography of diF-TES-ADT: PαMS blends to be less continuous and more defective as compared with the PS-based blend. This is probably the key reason for the lower performance from PαMS devices. Furthermore as the temperature dependence of viscosity of the polymer solution follows the Williams–Landel–Ferry model, the viscosity of polymer solutions is dependent on the glass transition temperature^64^. PS has lower glass transition temperature of (ca. 100 ^o^C) than PαMS (ca. 170 ^o^C), which is likely to yield higher viscosity of PS blend solutions at a coating stage temperature of 70 ^o^C. This may factor into the well connected, smooth and defect-free morphology of diF-TES-ADT films produced by blending with PS.

AFM micrographs taken of the surface of neat diF-TES-ADT and polymer blends are shown in Supplementary Fig. 5. For lower blading speed (0.5 mm s^−1^), the micrographs reveal significant crevices between adjacent ribbon-like crystals, whereas at higher speed (1.5mm s^−1^) all films show spherulite-like crystals connected by tall domain boundaries and exhibit significant topographic defects, such as gaps and cracks within the spherulites. Blending with PαMS or PS in the low-speed regime results in well-connected ribbons with topographically smoother boundaries. Blending with PαMS in the high-speed regime results in topographically smooth boundaries between spherulites as well, suggesting they are well connected, but still reveals significant topographic features and defects within the spherulites themselves. Blending with PS on the other hand leads to well-connected smooth spherulites with apparently smooth and continuous boundaries with fewer tomographic defects, hinting at the presence of morphological reasons for the better performance observed in PS-based blends over PαMS-based blends at higher blading speeds.

These results are remarkable considering that lamellar texture purity, a common challenge for spin-cast diF-TES-ADT, is crucial to achieving high carrier mobility^26^,^65^. In fact, spin-cast neat and blend solutions lead to diF-TES-ADT crystallization with a mixture of <001> and <111> textures exhibiting typical mobility of 10^−3^–10^−2^ cm^2^ V^−1^ s^−1^, unless special care is taken to chemically treat the bottom contacts^36^. Halogenated self-assembled monolayers are used to treat the surface of the gold electrodes to induce preferential growth of the <001> textured crystals during spin coating^26^,^65^,^66^. We show here that neither the neat OSC nor the OSC:polymer blend crystallize in any texture other than the <001> orientation whether PFBT treatment is used or not. This is proven by static grazing incidence wide-angle X-ray scattering (GIWAXS) images (Supplementary Fig. 6). We can see from this data taken in different processing conditions that only the <001> texture is formed by blade coating, irrespective of blending or not. This suggests a different nucleation and growth process may be operant in blade coating as opposed to spin coating. The reason behind this remarkable result is unknown and is beyond the scope of this study, but we speculate that this may be related to the blade-coating methods demonstrated confinement effect during crystallization of the OSC^67^. We also show in Supplementary Fig. 7 the μGIWAXS mapping data of neat diF-TES-ADT at 0.5 and 1.5 mm s^−1^ and diF-TES-ADT: PαMS (100 kDa) blend at 0.5 mm s^−1^ in Supplementary Fig. 7. We used two different X-ray beam incidence orientations of 0–90^o^ (0^o^ being defined as parallel to blade's moving direction). We can see that for the neat sample at 0.5 mm s^−1^, the (21L) peaks are present at 90^o^, while they disappear at 0^o^. The peaks are present in both orientations at 1.5 mm s^−1^. This indicates that the crystal growth is highly anisotropic at low speed, while at higher speed it is more isotropic. A similar observation is made for diF-TES-ADT: PαMS (100 kDa) at 0.5 mm s^−1^. This highlights the remarkable microstructure achievable using the blade-coating method and is likely to be at least partly responsible for achieving the typically higher carrier mobility even in neat films.

As we coat films by blade coating, it calls for an assessment of whether there are any shear stresses induced by the coating process, including in the presence of the binder polymers in the solution. PαMS and PS have different glass transition temperatures of 170 and 100 ^o^C, respectively, which calls for a closer evaluation of the difference in solution viscosities. We have performed rheological measurements on the starting solutions to investigate these effects. As all the coating experiments are performed in the speed range of 0.25–2 mm s^−1^ with a 100-μm gap between the substrate and the blade, the corresponding shear rate values of interest lie in the range from 2.5 to 20 s^−1^. In Supplementary Fig. 8a,b, we plot the shear stress and viscosity versus shear rate at room temperature for the blank solvent (toluene), diF-TES-ADT in toluene, and diF-TES-ADT blended with PS (Mw=123 kDa) and PαMS (Mw=100 kDa) using initial concentrations of the formulation. From these measurements, it is evident that there is no obvious difference between the shear stress and viscosity dependencies of the initial solutions on adding diF-TES-ADT and even after adding the binder polymers. Here a significant and uncontrolled evaporation of the solvent during the experiment can induce artefacts and makes it necessary to perform experiments in an enclosed set-up, which can be heated to the actual processing temperature of 70 ^o^C. However, we could not obtain any reliable data below a shear rate of 350 s^−1^. Nevertheless, the key takeaway from the rheological data obtained at higher temperature is that there is no apparent difference between the solution viscosities of the neat diF-TES-ADT solution and the blend solutions with PαMS and PS. This suggests that different glass transition temperatures of PS and PαMS are not affecting the initial solution viscosities at 70 ^o^C. We observe a shear thickening effect in the high shear rate regime of all three samples at elevated temperature, which is equivalent to blade-coating speeds nearly two orders of magnitude faster than the range of relevance to this study and may be interesting to investigate in the future. While our rheological analysis of the initial solutions does not reveal any meaningful differences in the shear stress and viscosity responses of the starting formulations, we do not discount the possibility that these parameters may be changing differently for the different formulations as the solution dries on the way to solid state thin-film formation. However, this is outside the scope of the current study.

### Influence of molecular weight of the insulating polymer

**Figure 3:**
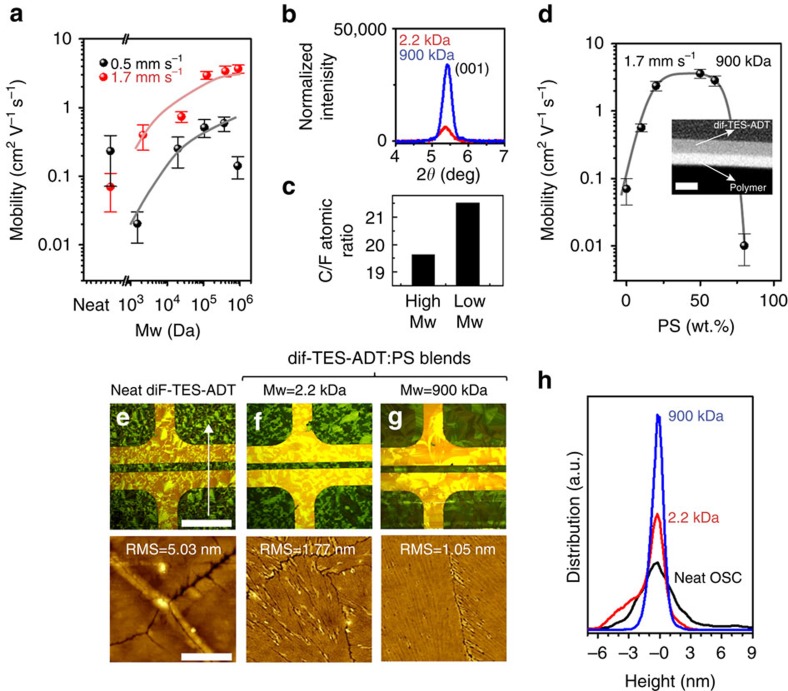
Molecular weight dependence of morphology and vertical phase separation. (**a**) Hole mobility of OTFTs fabricated by blade-coating diF-TES-ADT:PS blends using different Mw of PS both in the low-speed (0.5 mm s^−1^) and high-speed (1.7 mm s^−1^) regimes. (**b**) X-ray diffraction data of the (001) lamellar peak of diF-TES-ADT for blends prepared using low- (2.2 kDa) and high (900 kDa)-molecular weight PS at 1.7 mm s^−1^. Error bars in **a** and **b** represent s.d. from average values. (**c**) Atomic ratio of C and F obtained from XPS analysis of the surface of blends prepared using low- (2.2 kDa) and high (900 kDa)-molecular weight PS at 1.7 mm s^−1^. (**d**) Hole mobility of high-Mw blends with respect to the fraction of PS at 1.7 mm s^−1^. Inset shows a cross-sectional EFTEM micrograph of the bilayer stratification of the blend with PS at the bottom and diF-TES-ADT on top. Scale bar, 20 nm (in EFTEM image). Polarized optical micrographs (POM) and atomic force micrographs (AFM) of **e** neat diF-TES-ADT, (**f**) low-Mw blend and (**g**) high-Mw blend and white arrow shows the direction of blade coating. Scale bars, 250 μm (in POM images) and 4 μm (AFM images). (**h**) Statistical distribution of surface height obtained from AFM analysis of neat diF-TES-ADT, low-Mw blend and high-Mw blend.

The Mw of the polymer binder is known to influence the viscosity of the solution, as well as its solubility and miscibility^66^,^68^. It is consequently an important parameter to investigate and understand. In Fig. 3a, we have plotted the mobility as a function of the Mw of PS for diF-TES-ADT:PS (1:1) blends. In the low-speed regime, blending the OSC with low-Mw PS markedly reduces the mobility, whereas increasing the Mw beyond 100 kDa yields mobility as high as ∼4.5 cm^2^ V^−1^ s^−1^, far superior to the neat sample. POM images of the low-speed samples (Supplementary Fig. 9) indicate the ribbon formation is disrupted by blending with low-Mw PS, whereas it is not disrupted by high Mw. This is also confirmed by AFM analysis (Supplementary Fig. 3). In Fig. 3b, we have plotted the lamellar (001) X-ray diffraction peak for diF-TES-ADT for the low- and high-Mw blends, normalized to film thickness estimated by ellipsometry. It is clear that lamellar crystallinity is substantially improved by OSC blending with the high-Mw polymer. We have also plotted in Fig. 3c the atomic ratio of carbon and fluorine extracted from X-ray photoelectron spectroscopy (XPS) analysis of the top surface of blends prepared using low- and high-Mw PS at low blading speed. The ratio is expected to be 19.6 for the pure OSC. However, the analysis reveals lower fluorine/higher carbon content in the low-Mw case, indicating the presence of some PS near the exposed surface of the blend. By contrast, the high-Mw blend seems to have an OSC-rich surface, consistent with the formation of a bilayer with the OSC near the top. These results clearly indicate the Mw of PS strongly influences the vertical stratification of the blend components, which is believed to promote or disrupt long-range lateral crystallization of the OSC. This may be related at its origin to differences in phase separation behaviour in the presence of low- and high-Mw blends, which can exhibit Mw-dependent solubility and miscibility^38^,^39^,^69^,^70^.

We achieve a remarkable boost in performance when casting the blend in conditions of higher blading speed (Fig. 3a). The carrier mobility increases steadily with increasing Mw, rising to as high as 4.5 cm^2^ V^−1^ s^−1^. The device figures of merit include on/off >10^6^ and threshold voltage of −0.1 V. The remarkable mobility can be understood by examining and comparing POM and AFM images of the neat film (Fig. 3e) to the low-Mw (Fig. 3f) and high-Mw (Fig. 3g) blends. The micrographs reveal the formation of substantially larger domains in the high-Mw case than in neat OSC or low-Mw blends. The best films appear to be topographically smoother, are crack-free and appear to have sharp domains and grain boundaries, as indicated by AFM. Statistical distributions of the surface height were calculated from the entire AFM scan area (10 × 10 μm) and summarized in Fig. 3h. The distributions have been centred at an arbitrary height of zero since the AFM tip does not sense the substrate in either case. From the shape and width of the distribution, it is quite clear that the neat film is the roughest sample (*σ*_r.m.s._ ∼5 nm; r.m.s, root mean squared) with features both tall and deep. The low-Mw blend (*σ*_r.m.s._ ∼1.8 nm) has a significant hump on the deep side, indicating that the film contains pin holes, cracks or deep valleys at grain boundaries. By contrast, the high-Mw blend appears to show a very narrow and symmetric distribution (*σ*_r.m.s._ ∼1.1 nm), consistent with a very smooth OSC surface free of pin holes, cracks and exhibiting closed grain/domain boundaries. Polymer solution viscosity is strongly dependent on Mw of polymers and increases with increasing Mw of polymer. The significant differences observed from the morphological investigations suggest that higher solution viscosities of high-Mw PS blend solutions are possibly factoring into the formation of crack-free and topographically smooth domains of tightly connected OSC domains.

We have imaged the vertical phase separation of the optimal high-Mw blend film prepared at 1.5 mm s^−1^ using cross-sectional EFTEM (Fig. 3d, inset). The micrograph clearly shows a bilayer phase separation with the OSC on top and the PS layer sandwiched between the OSC and the SiO_2_ dielectric. The total thickness of the bilayer is found to be ∼21 nm with sublayer thicknesses of ∼11 and ∼10 nm for the OSC and PS, respectively. We also performed similar analysis for samples prepared at 0.5 and 1.0 m ms^−1^ (Supplementary Fig. 10) to confirm that vertical phase stratification is similar at different speeds. These observations are in agreement with the recent report of BCBG OTFTs of diF-TES-ADT:PS in which the blend film has been shown to undergo bilayer vertical phase stratification as characterized by TEM^41^.

In the case of low-Mw PS, we observe a mixed phase of OSC and polymer on top as seen from XPS results in Fig. 3b. By contrast, the high-M_w_ PS case yields a PS-free surface as confirmed by cross-sectional EFTEM analysis as well (Fig. 3d). The above results clearly highlight the critical role the Mw of the insulating polymer plays in promoting long-range lamellar order via effective phase separation and vertical stratification. They also marginalize the low-Mw polymer as a poor candidate for high-performance blend OTFTs^35^,^44^.

Our investigation of blading speed and polymer Mw has been restricted thus far to a 1:1 w/w blend ratio of the OSC and polymer. In Fig. 3d, we plot the mobility with respect to the blend ratio of OSC:PS for a fixed Mw of 900 k to assess the compositional window over which the blending scheme is effective at promoting vertical stratification for a fixed overall solute concentration. The plot reveals three distinct regions: a PS-deficient device regime for [PS]<20%, a stable high-performance regime for a composition 20%<[PS]<60% and an OSC-deficient regime for [PS]>60%. Approaching either extreme appears to disrupt the uniform surface morphology or crystalline domains as revealed by POM and AFM (Supplementary Fig. 11a). Both extremes lead to smaller and distinctive crystallites of the OSC with high density of grain boundaries and cracks, whereas the stable performance regime, including 50% PS, is characterized by very large and well-connected domains with few gaps, cracks and surface morphological features visible to either AFM or POM. It seems that even a small amount of PS can have significant benefits with respect to device operation. If we look at the transfer curves and figures of merits such as *V*_th_ and SS (Supplementary Fig. 11b) for devices with very low PS content (10% PS), we find that *V*_th_ decreases substantially from 40 V (neat) to 15 V. Continuing to add PS, *V*_th_ decreases to 2.5 V for [PS]=20% and 0.4 V when [PS]=50%. Meanwhile, SS improves from 7 (neat) to 2.5 (10% PS), 0.55 (20% PS) and 0.3 V dec^−1^ (50% PS). We have calculated *N*_it_ for these cases and summarized it in Table 2. *N*_it_ decreases gradually from ∼10^13^ eV^−1^ cm^2^ in the neat OSC case to 2.7 × 10^12^ eV^−1^ cm^2^ with 10% PS and 2.65 × 10^11^ eV^−1^ cm^2^ with 50% PS. These remarkable improvements suggest that addition of PS to the diF-TES-ADT solution leads to significant improvements to the OSC–dielectric interface and may possibly affect the OSC–contact interface as well.

### Influence of solvent mixtures

**Figure 4:**
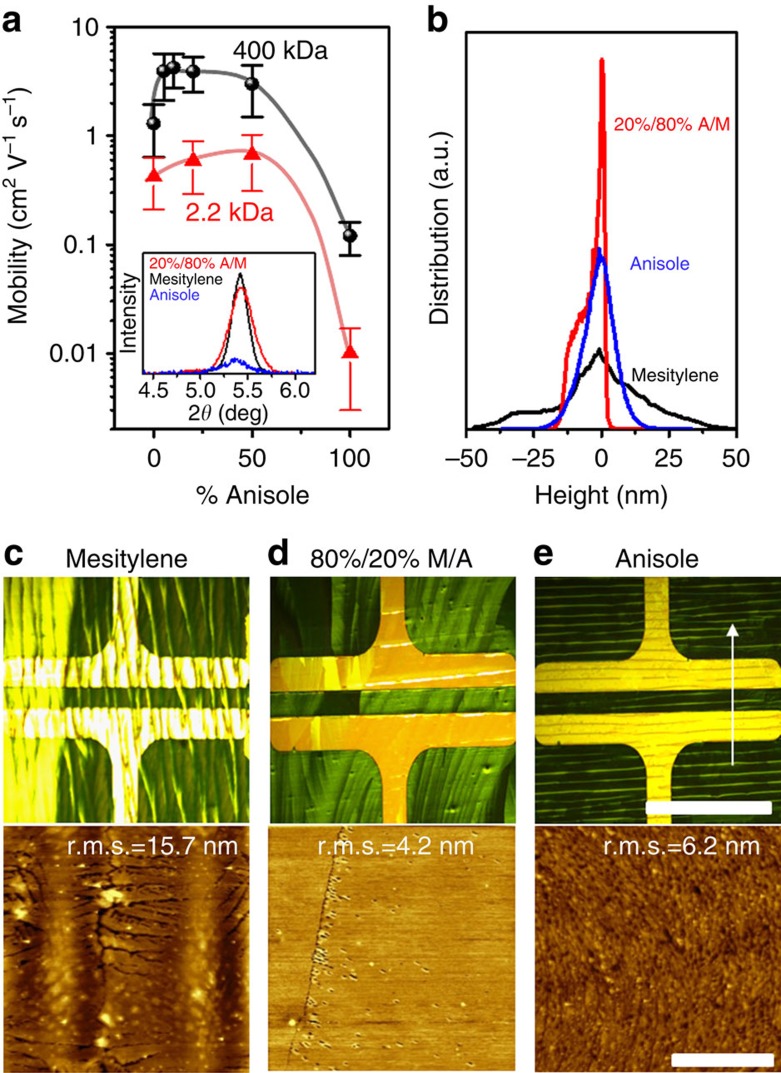
Impact of solvent mixtures on OTFT performance and morphology. (**a**) Mobility with respect to the volume fraction of anisole in the anisole/mesitylene solvent mixture for diF-TES-ADT:PS blend OTFTs using low- (2.2 kDa) and high (400 kDa)-Mw PS. The inset shows X-ray diffraction data around the (001) lamellar diffraction peal of diF-TES-ADT comparing pure anisole, pure mesitylene and 20%/80% anisole/mesitylene mixtures. Error bars in a represent s.d. from average values. (**b**) Statistical distributions of surface height extracted from AFM data of mesitylene, anisole and 20%/80% anisole/mesitylene solvent mixture. POM and AFM images of blends based on diF-TES-ADT:PS (Mw=400 kDa) using (**c**) mesitylene, (**d**) 20%/80% anisole/mesitylene mixture and (**e**) anisole and white arrow shows the direction of blade coating. Scale bars, 500 μm (in POM); 20 μm (AFM images).

Blending diF-TES-ADT with the high-Mw PS has led to a remarkable ∼50 × improvement in carrier mobility and marked improvements of other OTFT figures of merit. However, the carrier mobility of the films is still inferior to what has been achieved by spin coating of semiconducting polymer:OSC blends (5.4 cm^2^ V^−1^ s^−1^) or by single crystals of the OSC (6 cm^2^ V^−1^ s^−1^) (refs 49, 50). Close inspection of POM images of the PS:OSC blends in actual devices reveals that the domain size is smaller than the area of a single device (Fig. 3g), hinting that better results might be achieved if more could be done to extend the domain and grain sizes. We recall a previous study in which a dual-solvent approach was used consisting of a mixture of polar and non-polar solvents to increase the size of OSC domains in neat films^48^. We take the view that the appropriate solvent mixture can enhance polymer solubilization, for example, in the main solvent, and lead to enhanced phase separation, which might further promote the in-plane growth of lamellar OSC sheets. We have selected anisole and mesitylene as the polar and non-polar (main) solvents, respectively, and varied their mixing ratio. To explain the rationale for this choice, we consider the Hansen solubility parameters for anisole, mesitylene, toluene and PS (Table 3; *δ*d, *δ*p and *δ*h represent the energy associated with dispersion, dipolar and hydrogen-bonding forces, respectively). On the basis of these parameters, anisole is a better solvent for PS, while mesitylene is a poor solvent. Furthermore, we confirm the differences in solubility of diF-TES-ADT in both solvents and in mixtures thereof by performing ultraviolet–visible (ultraviolet–vis) absorption measurements of dilute diF-TES-ADT:PS blend solutions (ca. 0.5 mg ml^−1^) as shown in Supplementary Fig. 12. The addition of anisole in the blend solution causes a red shift, which indicates an increase in the aggregation of diF-TES-ADT. This shift also suggests that anisole is a poorer solvent for diF-TES-ADT than mesitylene^71^,^72^. Possibly, these two solvents can also form an azeotropic binary solvent mixture (different solubility of the solute in binary solvent mixture), which has been proven to be beneficial to grow single crystals of TIPS:pentacene^73^. In the solid state, we observed a clear transition from ribbon-like crystallites (mesitylene) to sheet-like crystallites (mesitylene:anisole). This transition is consistent with what has been reported for Tips-pentacene using the binary azeotropic mixture of toluene/isopropanol (ref. 73). In Fig. 4a, we plot the hole mobility of OSC:PS blends prepared in different solvent mixtures using both low- and high-Mw PS as binder. All films were blade coated at a lower speed of 0.5 mm s^−1^ using a base temperature of 100 °C. Similar trends can be seen with respect to Mw as with single-solvent devices. High-Mw blends consistently outperform low-Mw ones by nearly an order of magnitude. This trend appears to hold across solvent mixtures. Importantly, we find that devices behave rather poorly in the case of pure solvents, whereas solvent mixtures, especially those rich in mesitylene (poor in anisole), behave particularly well. Pure anisole and mesitylene yield mobilities on the order of ∼0.1 and ∼1 cm^2^ V^−1^ s^−1^, respectively. We achieve the highest hole mobility of 6.7 cm^2^ V^−1^ s^−1^ when the solvent mixture is 20% anisole/80% mesitylene.

The solvent mixture appears to form higher quality films than the single-solvent (anisole or mesitylene) cases. The solvent mixture leads to significantly higher lamellar crystallinity as indicated by X-ray diffraction measurement shown in the inset of Fig. 4a. However, the higher-order Bragg peaks of these very thin films (Supplementary Fig. 13) are extremely weak and do not show up in the X-ray diffraction analysis. We have highlighted the blade movement direction on all POM images with an arrow. The POM taken *in situ* during blade coating (Supplementary Fig. 14) shows the lines forming in the direction perpendicular to the blade movement. These lines are believed to be due to fluid dynamic instabilities commonly observed during blade coating^74^. We also observe by POM remarkably large domains spanning the millimetre scale (Fig. 4d). Closer examination by AFM reveals few if any topographic defects and boundaries (Fig. 4d). In the pure mesitylene case (0% anisole), ribbon-like crystalline features with significant cracking are observed by POM and AFM in Fig. 4c, whereas films cast from pure anisole result in the formation of very small grains (Fig. 4e). The statistical distribution of surface height extracted from the full AFM scan (50 × 50 μm) reported in Fig. 4b which is quite revealing in that it shows once more the remarkable flatness of the blend prepared by using a solvent mixture (*σ*_r.m.s._ ∼4.57 nm) as opposed to the single-solvent cases. We observe significant height variations (*σ*_r.m.s._ ∼15.71 nm) associated with grain and domain boundary features between ribbons in the case of pure mesitylene and smaller but still significant height variations (*σ*_r.m.s._ ∼6.24 nm) due to the formation of fine crystallites in the pure anisole case. Comparison between the (001) lamellar Bragg peak intensities of the best dual-solvent blend and the best single-solvent blend based on toluene (Supplementary Fig. 15) suggests a further improvement in the lamellar stacking quality obtained by the use of the solvent mixture. We therefore find mounting evidence suggesting that an improvement in lamellar crystalline quality and texture coupled with a flat, pinhole and crack-free OSC film with few topographically visible domain boundaries have the potential to yield carrier mobilities approaching and even on par with those of single-crystal devices of the same OSC.

**Figure 5:**
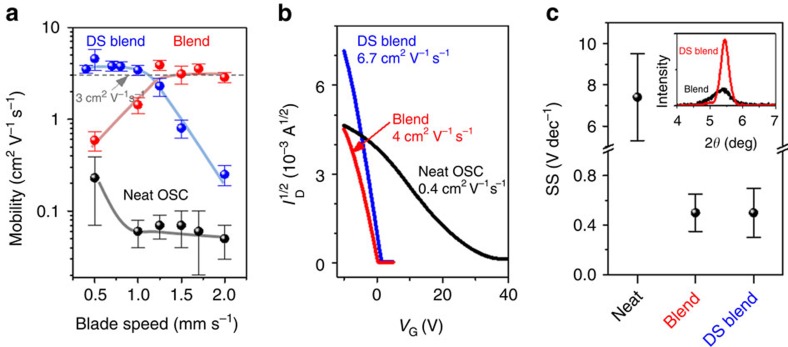
Single and dual-solvent blend OTFTs. (**a**) Mobility with respect to blade-coating speed for neat diF-TES-ADT as well as blends of diF-TES-ADT:PS (Mw=400 kDa) prepared using toluene (blend) and dual-solvent (DS blend) approaches. (**b**) *I*_d_–*V*_g_ curves showing transfer characteristics of the best performing BCBG devices prepared using the three approaches. We employed *V*_ds_=−10 V for blends and *V*_ds_=−20 V for neat OSC. (**c**) Subthreshold swing obtained using the three approaches. The inset shows X-ray diffraction data around the (001) lamellar diffraction peak of diF-TES-ADT in blends prepared using toluene and the dual-solvent mixture. Error bars in **a** and **c** represent s.d. from average values.

The record-breaking dual-solvent blend was cast at a lower blading speed (0.5 mm s^−1^) than the optimal speed for the single-solvent (toluene) case, suggesting that changes in formulation can help to maintain high performance across a broad window of coating conditions. We have summarized in Fig. 5a, the blade-coating speed dependence of device performance for neat OSC as well as blends based on the single-solvent (toluene) and dual-solvent (anisole/mesitylene) approaches. This comparison shows that a broad processing window exists within which consistently high carrier mobility (>3 cm^2^ V^−1^ s^−1^) can be achieved simply by adjusting the formulation. By contrast, neat films prepared using toluene typically yield mobility <0.1 cm^2^ V^−1^ s^−1^.

We compare in Supplementary Fig. 16 the morphological features of the neat OSC and blends prepared using toluene and dual-solvent approach obtained in conditions yielding the best OTFT devices for each approach. Neat diF-TES-ADT forms ribbon-like-oriented crystals with large crevices between adjacent ribbons, while the blends yield much smoother films with largely isotropic domains. The single-solvent blend approach yields polycrystalline OSC films with several large domains visible under POM and well connected as evidenced by AFM. The dual-solvent blend approach shows no distinctive domains under POM and yields films with little if any boundaries on the scale of the OTFT device. This absence of any microstructurally distinct domains in the dual-solvent case yields ultrathin films, which appear single-crystal-like in many ways and certainly yield performance on par or even surpassing state-of-the-art single-crystal FET devices made from the same OSC.

In summary, we demonstrate remarkable OTFTs using a blade-coating method potentially compatible with continuous roll-to-roll manufacturing. We blended the OSC with high-Mw amorphous insulating polymers to achieve this result and further explored solvent mixtures to ultimately fabricate devices with performance on par with or even surpassing single-crystal FETs based on the same OSC. Using a combination of polarized optical microscopy, atomic force microscopy, X-ray diffraction and cross-sectional EFTEM, we demonstrated that the keys to the remarkable OTFT performance are the formation of a bilayer stratification with an ultrathin ∼10-nm-thick single-crystal-like OSC layer on top and the polymer at the bottom, formation of millimetre scale large coherent domains with few boundaries and with very smooth, pinhole- and crack-free topography of the OSC surface both within the domains and at their boundaries. The devices featured herein achieve consistently high carrier mobility even over a broad range of processing conditions and exhibit exceptional figures of merit, such as on–off ratio exceeding 10^6^, low threshold voltage (0.1 V) and low subthreshold swing (0.3 V dec^−1^), making them suitable for implementation in high-performance flexible displays and circuits.

## Methods

### Device fabrication and characterization

BCBG organic OTFTs were fabricated on Si/SiO_2_ substrate having 300 nm SiO_2_ as dielctric and gold electrodes of 50-nm thickness. Devices had channel lengths and widths of 80μm and 1 mm, respectively. diF-TES-ADT, PS and PαMS were dissolved in toluene with a concentration of 10 mg ml^−1^. The OSC and polymer solutions were stirred for 1 h before mixing them together in 1:1 w/w ratio. The resulting neat diF-TES-ADT and blend solutions were blade coated using a set-up described in detail elsewhere^11^,^20^,^65^. The blading speeds explored range between 0.5 and 2 mm s^−1^, by 0.25 mm s^−1^ steps at a fixed stage temperature of 70 ^o^C for the single-solvent approach using toluene. For the dual-solvent approach, blend solutions were made using 1:1 w/w of diF-TES-ADT and PS in mesitylene and anisole and then these two solutions were mixed together in V/V ratios. The films were removed quickly from the hot plate to prevent any loss of initial phase separation or dewetting. Electrical measurements were performed in a nitrogen atmosphere using a semiconductor parameter analyser (Kiethley 4200 SCS). Field-effect mobilities were calculated using the standard thin-film model in saturation regime of the device using 
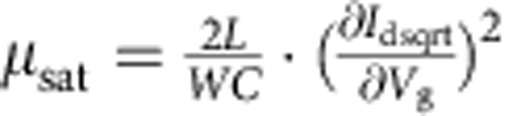
, where *L*, *W* and *C* are the channel length, channel width and geometric capacitance of the dielectric, respectively. We used −10 V=*V*_ds_ for all blend devices and −20 V=*V*_ds_ for neat devices. We used the effective geometric capacitance of the dielectric by incorporating the insulating polymer layer thickness and dielectric constant in the calculation (Supplementary Fig. 17).

### Electron microscopy

A transmission electron microscope operating at 300 kV (Titan Cryo Twin, FEI Company, Hillsboro, OR) was used to acquire cross-sectional microscopy images using a 4 × 4k charged-couple device camera model US4000 and an energy filter model GIF Tridiem from Gatan, Inc. (Gatan Inc., Pleasanton, CA). EFTEM images show carbon maps that are generated using the so-called ‘jump-ratio' method. First, we acquired the post-carbon-edge (C-K edge) image by shifting the electron energy to 292 eV and using energy slit width of 20 eV. In a second step, the pre-carbon edge image was acquired by shifting the electron energy to 272 eV and using the same slit width of 20 eV. The resultant carbon jump-ratio map was obtained by dividing the post-edge image with the pre-edge image. Samples (size 12 × 4 μm) were prepared using a focused ion beam (Helios 400s, FEI) equipped with a nanomanipulator (Omniprobe, AutoProbe300) with lift-out method. The sample was sputter coated with a 22-nm-thick Au layer to prevent charging effects. E-beam and ion beam-assisted Pt deposition were subsequently deposited to protect the surface of the sample against Ga ion damage during ion beam milling. GA ion beam milling was first used to cut the sample from the bulk (30 kV, 9 nA), after which it was attached to a Cu grid using a lift-out method. The sample was subsequently thinned down (30 kV, 0.98 nA) and cleaned (2 kV, 28 pA) to get rid of areas of the sample damaged during the thinning process.

### X-ray diffraction experiments

X-ray diffraction measurements in the *θ*−2*θ* mode with 2*θ* ranging from 4° to 20° on a Bruker X-pert diffractometer. The beam slits were set to 1 × 1 mm.

### High-speed polarized optical microscopy and atomic force microscopy

High-speed POM was performed on Nikon LV-100 microscope and recorded using a Photron SA-3 CMOS camera. The surface topography was evaluated using the Agilent 5400 SPM operating in tapping mode. Image planarization and statistical analyses were performed using Gwyddion V2.25.

### Ultraviolet–vis absorption experiments

The ultraviolet–vis absorption measurements were performed using a F20-UVX spectrometer (Filmetrics, Inc.) equipped with tungsten halogen and deuterium light sources (Filmetrics, Inc.) over the wavelength range of interest, from 400 to 800 nm.

### Rheological measurements

Rheological measurements were performed using an Anton Paar MCR-501 rheometer with a cone plate system (CP25-1) for testing at 25 °C and a double-gap module (DG35.12/pr) for testing at 70 °C. As the CP25-1 system is open, it cannot prevent solvent loss during measurements at 70 °C due to solvent (toluene) evaporation. For that reason, we have used the closed system (double-gap module DG35.12/pr) at 70 °C to prevent solvent loss during measurements. A shear rate from 2 to 5,000 s^−1^ was applied for all tests and the slope was set as 20 points per decade.

## Additional information

**How to cite this article:** Niazi, M. R. *et al*. Solution-printed organic semiconductor blends exhibiting transport properties on par with single crystals. *Nat. Commun.* 6:8598 doi: 10.1038/ncomms9598 (2015).

## Supplementary Material

Supplementary Information Supplementary Figures 1-17

## Figures and Tables

**Table 1:** Interfacial trap state density for neat and blended OTFTs.

**Trap density (eV**^−1^** cm**^2^)	**Neat diF-TES-ADT**	**diF-TES-ADT:PαMS**	**diF-TES-ADT:PS**
*N*_it_	8.2 × 10^12^−4.6 × 10^13^	4.5 × 10^12^−1 × 10^12^	3.2 × 10^11^−6.5 × 10^11^

diF-TES-ADT, 2,8-difluoro-5, 11-bis(triethylsilylethynyl) anthradithiophene; OTFT, organic thin-film transistor; PS, polystyrene; PαMS, poly(alpha-methyl styrene).

**Table 2:** Interfacial trap state density for diF-TES-ADT:PS blend OTFTs as a function of PS concentration.

**Trap density (eV**^−1^** cm**^2^**)**	**0% PS**	**10% PS**	**20% PS**	**50% PS**
*N*_it_	3 × 10^13^	2.69 × 10^12^	5.4 × 10^11^	2.65 × 10^11^

diF-TES-ADT, 2,8-difluoro-5, 11-bis(triethylsilylethynyl) anthradithiophene; OTFT, organic thin-film transistor; PS, polystyrene.

**Table 3:** Hansen solubility parameters for the various solvents and polystyrene.

**Materials**	***δ*****d (MPa)**^**1/2**^	***δ*****p (MPa)**^**1/2**^	***δ*****h (MPa)**^**1/2**^
Polystyrene	18.5	4.5	2.5
Mesitylene	18	0.6	0.6
Anisole	17.8	4.4	6.9
Toluene	18.1	1.4	2.0
