# Supplementary material for: Solution-printed organic semiconductor blends exhibiting transport properties on par with single crystals
Source: Nat Commun. 2015 Nov 23;6:8598. doi: 10.1038/ncomms9598 (PMC4673501; doi:10.1038/ncomms9598)
Supplement: Supplementary Information — Supplementary Figures 1-17 [file ncomms9598-s1.pdf]

Supplementary Figures

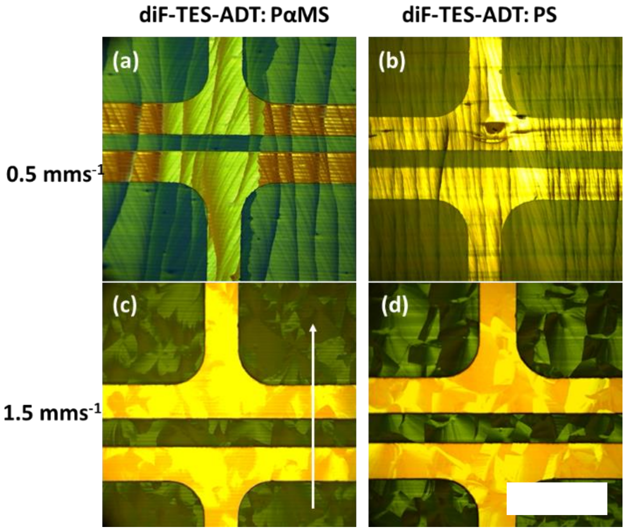

Supplementary Figure 1: POM images showing the ribbon like crystal formation at low blade coating speed and spherulites formation at high speed for diF:PαMS and diF: PS blend cases. The blade movement is shown by white arrow. The scale bar is 300 µm.

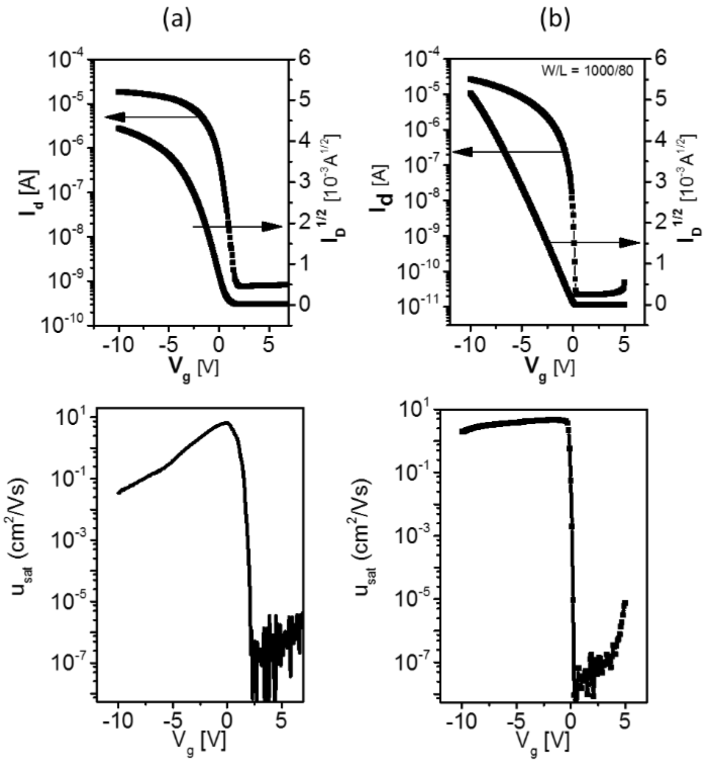

Supplementary Figure 2: Transfer characteristics and gate voltage dependence of hole mobility in BGBC OTFTs subjected to different surface cleaning procedures: (a) UV ozone cleaning (b) O_2-plasma cleaning. The plasma cleaning is much more effective and yields a nearly ideal transfer characteristics, which are required for reliable extraction of saturation hole mobility.

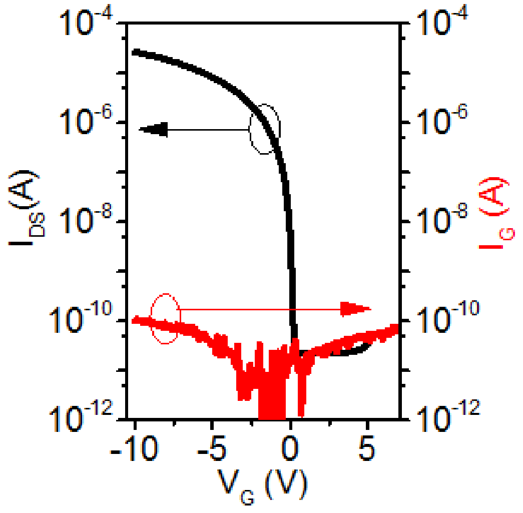

Supplementary Figure 3: Current vs voltage curves showing the transfer curves and gate current at Vds = -10 V.

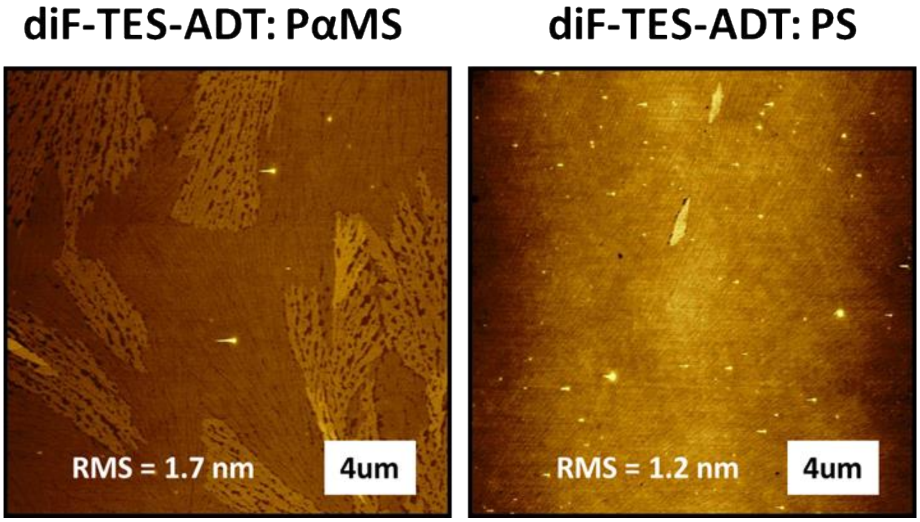

Supplementary Figure 4: Atomic force microscopy images of diF-TES-ADT:PαMS and diF-TES-ADT:PS blends prepared at blade coating speed of 2 mms^{-1} and substrate temperature of $70^{o}C$. The images hint at why PS-based blends perform better than PaMS-based ones.

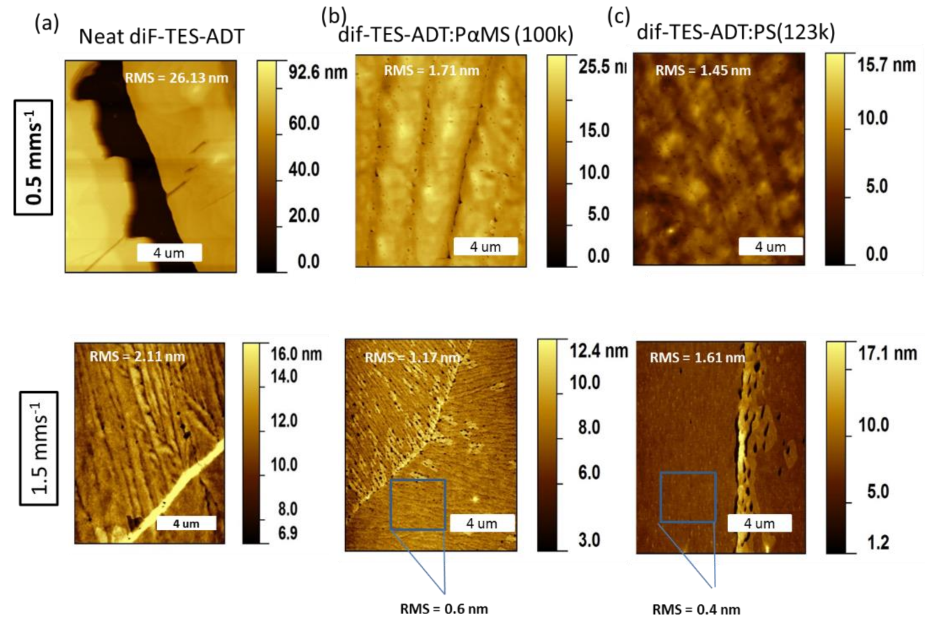

Supplementary Figure 5: AFM topographic images for (a) neat diF-TES-ADT (b) diF: PαMS and (c) diF: PS blends at low and high blade speeds.

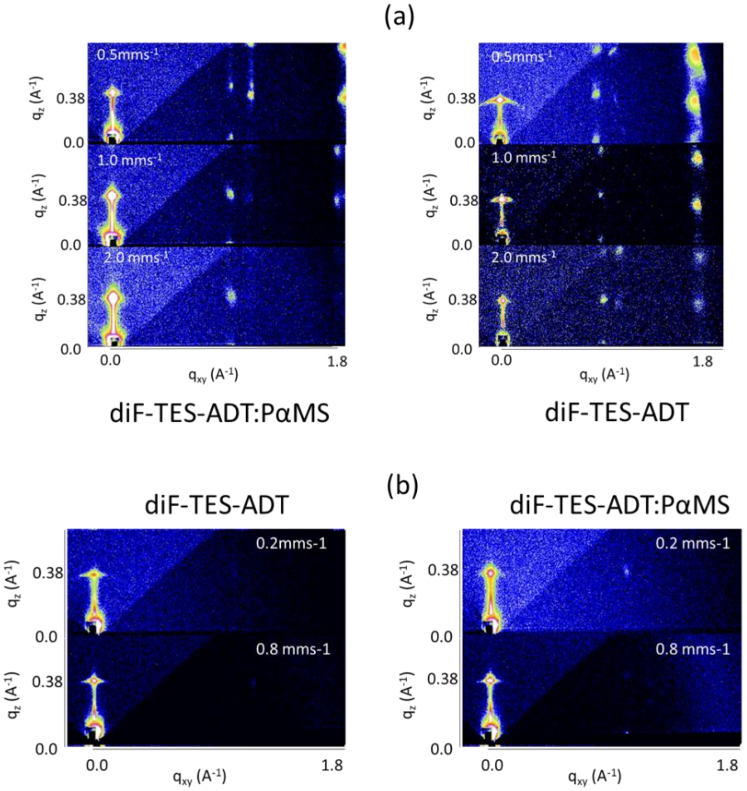

Supplementary Figure 6: Static GIWAXS images for diF: PαMS and neat diF-TES-ADT at different speeds showing the existence of only pure <001> texture (a) samples were prepared at blading speed of 0.5, 1.0 and 2.0 mms^{-1} and substrate temperature of 70°C (b) samples were prepared at blading speed of 0.2 and 0.8 mms^{-1} and substrate temperature of 25°C.

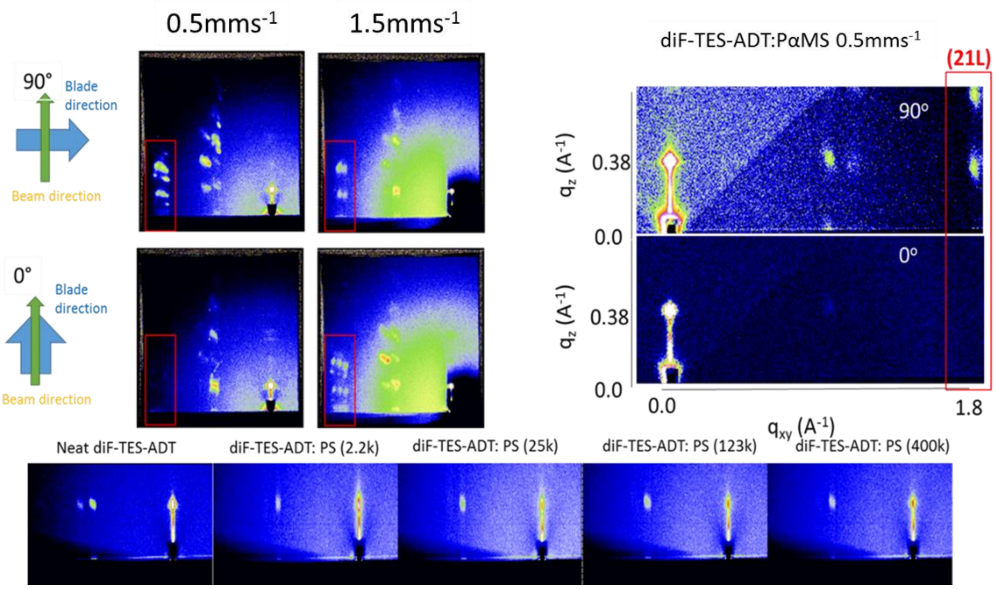

Supplementary Figure 7: (top) GIWAXS patterns of neat diF-TES-ADT and blend films with the beam perpendicular and parallel to the casting direction. The 0° angle is defined as the x-ray beam parallel to the blading direction. (bottom) GIWAXS patterns for neat diF-TES-ADT and blends with different Mw of PS as highlighted in the figure. Note: The red box indicates the (21L) peaks.

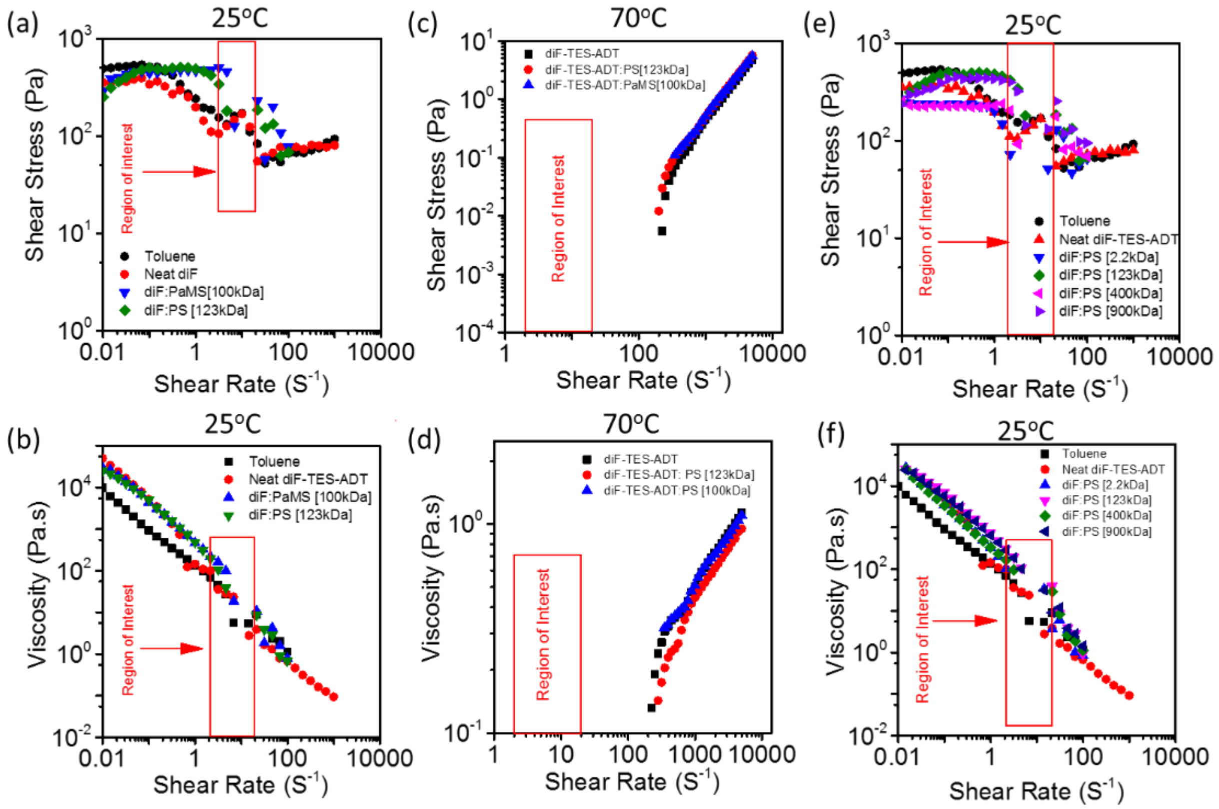

Supplementary Figure 8: Plots of (a) & (c) shear stress *vs.* shear rate and (b) & (d) viscosity *vs.* shear rate for the blank solvent, neat diF-TES-ADT solution, diF-TES-ADT:PS (Mw = 123 kDa) and diF-TES-ADT:PαMS (Mw= 123 kDa) blend solutions at 25°C and 70°C (temperature indicated on the plot). Plots of (e) shear stress *vs.* shear rate and (f) viscosity *vs.* shear rate for the blank solvent, neat diF-TES-ADT solution and its blends with PS (different Mw).

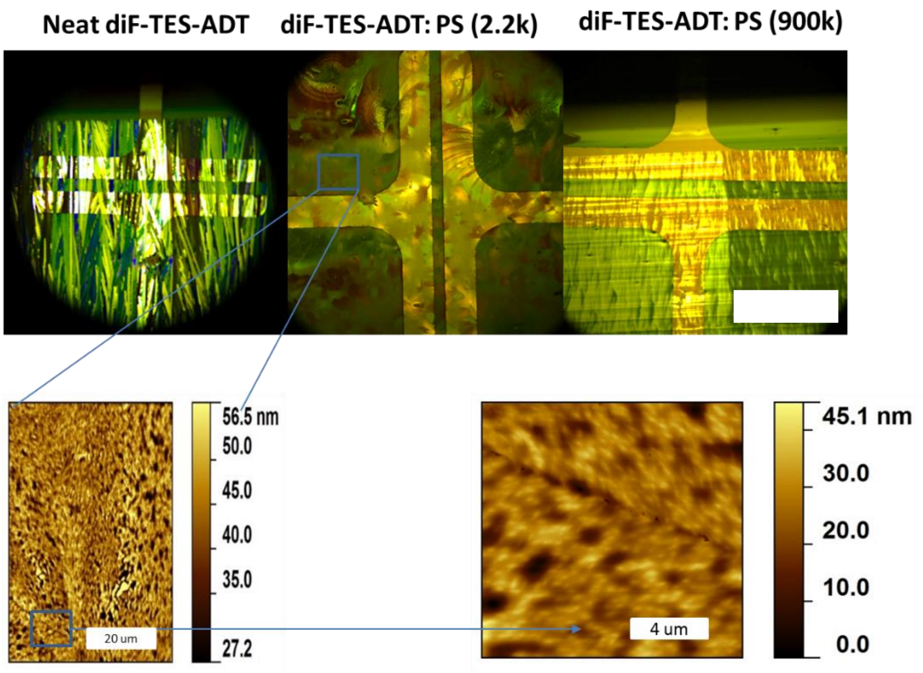

Supplementary Figure 9: Images taken from in situ high-speed POM videos and corresponding AFM images showing the disruption of regular crystal growth process in case of low Mw PS: diF-TES-ADT blend at 0.5mms^{-1}. The scale bar is 300 µm.

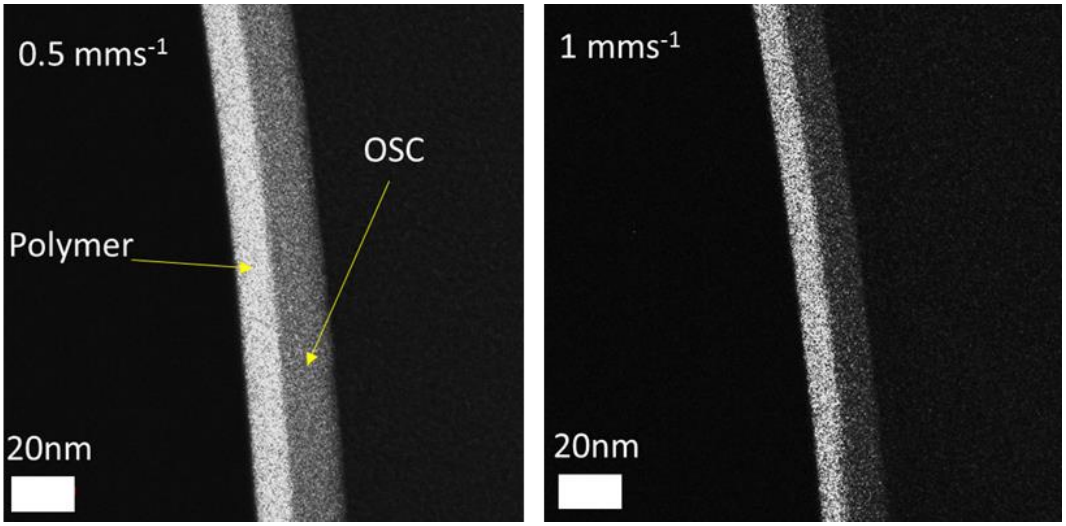

Supplementary Figure 10: Cross section EF-TEM images of diF-TES-ADT: polymer blends prepared at 0.5 and 1.0 mm s^{-1} at 70°C. Contrast obtained using carbon jump ratio.

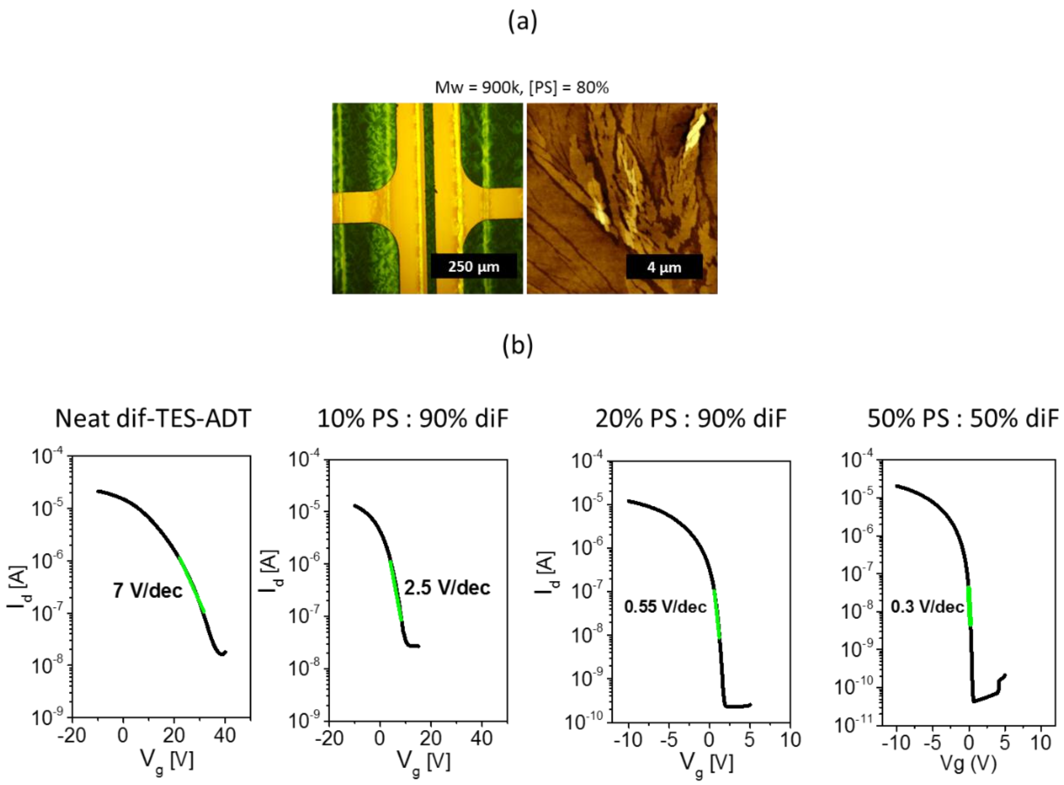

Supplementary Figure 11: (a) POM and AFM micrographs of 80% PS:20 diF-TES-ADT sample (b) Transfer charcteritics of BCBG OTFTs corresponding to different blend ratios of PS (900k) with diF-TES-ADT, highlighting the subthreshold swing decrease.

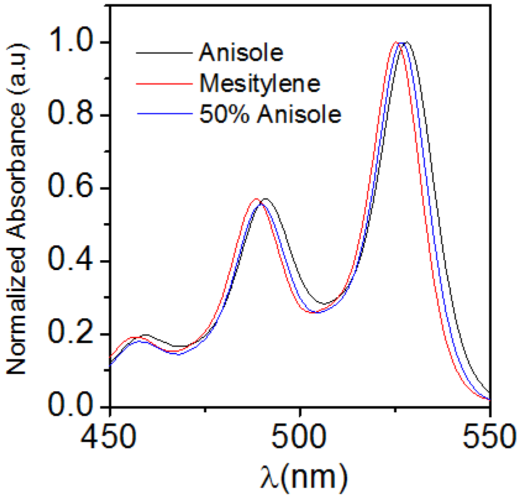

Supplementary Figure 12: UV-Vis absorptions spectrum for diF-TES-ADT:PS [400kDa] in mesitylene, anisole and their mixture

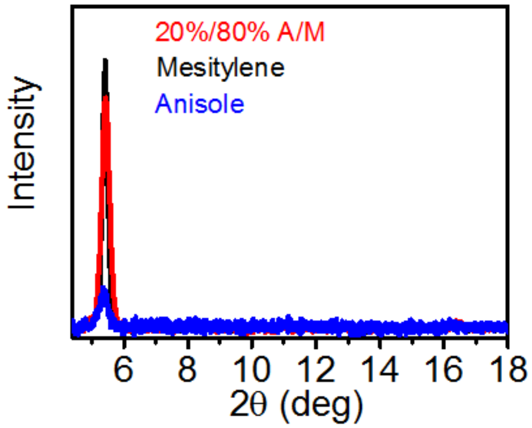

Supplementary Figure 13: XRD spectra for diF-TES-ADT: PS blend in pure and dual solvent mixture showing the extended range of 2θ.

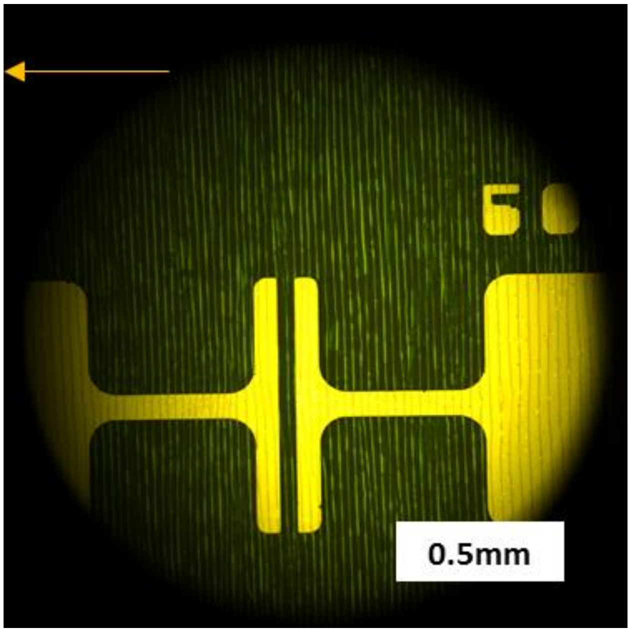

Supplementary Figure 14: Polarized optical micrograph taken during blade coating and showing the crystallization behavior of diF-TES-ADT: PS blends in 100% Anisole (Note: The arrow shows the direction of blade coating and therefore the vertical lines are not related to crystallographically oriented ribbon formation

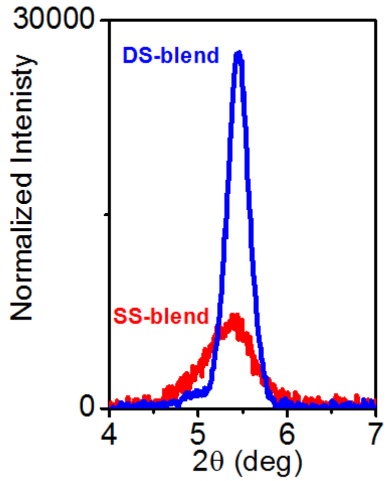

Supplementary Figure 15: XRD (001) Bragg peak showing the difference between the best single solvent blend film and the best dual solvent blend film.

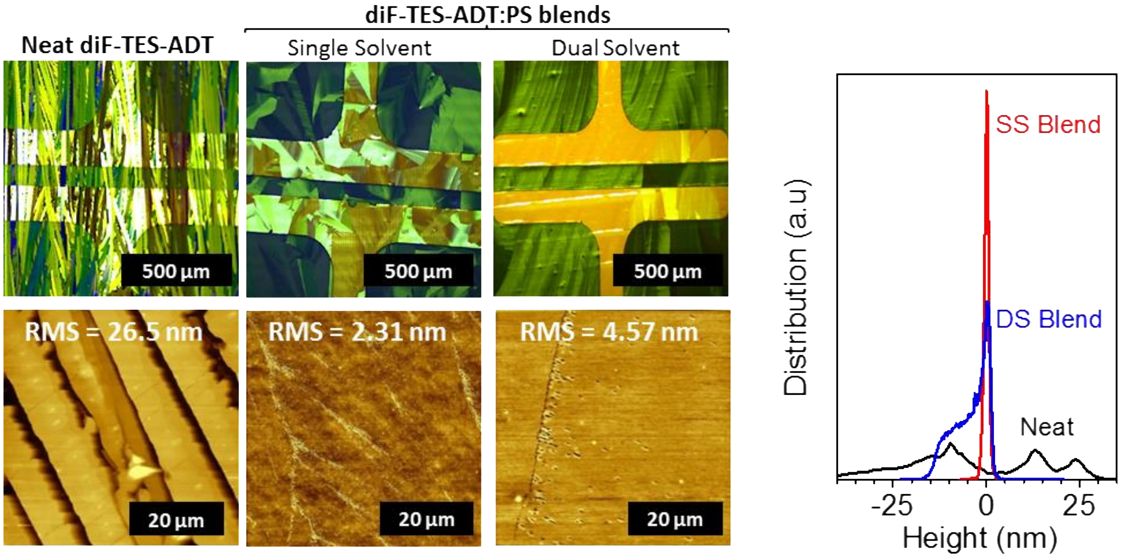

(a) **(b)**

Supplementary Figure 16: (a) POM and AFM images for best neat diF-TES-ADT, diF:PS single solvent blends and diF: PS dual solvent blend. (b) Height distribution profiles for neat diF-TES-ADT, blend in toluene and dual solvent mixture extracted from AFM topographic images.

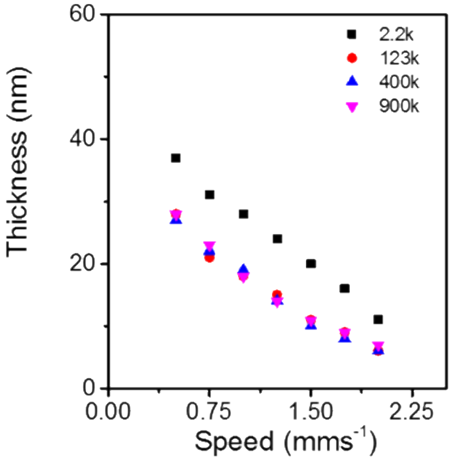

(a)

Speed (mms^{-1})	PS 2.2kDa	PS 123kDa	PS 400kDA	PS 900kDA	PaMS (100kDa)
0.5	9.44	9.78	9.82	9.78	9.90
0.75	9.66	10.06	10.02	9.98	10.18
1.0	9.78	10.18	10.14	10.18	10.27
1.25	9.94	10.31	10.35	10.35	10.39
1.5	10.10	10.48	10.53	10.48	10.53
1.75	10.27	10.57	10.62	10.57	10.62
2.0	10.48	10.71	10.71	10.66	10.76

(b)

Supplementary Figure 17: (a) Thickness of polystyrene layer buried under OSC with respect to blade speed as determinded by spectroscopic ellipsometery after selectively dissolving the OSC in cyclohexane. (b) Effective gate dielctric capacitance per unit area (nF cm^{-2}) for PS and PαMS at different speeds.
